# Vitamin E Enhances Cell Viability and the Osteogenic Differentiation of Cell Spheroids Made of Gingiva-Derived Stem Cells

**DOI:** 10.3390/medicina59040736

**Published:** 2023-04-09

**Authors:** Jin-Hyeong Kim, Minji Kim, Somyeong Hwa, Youngkyung Ko, Jun-Beom Park

**Affiliations:** 1 Department of Periodontics, College of Medicine, The Catholic University of Korea, Seoul 06591, Republic of Korea; 2 Dental Implantology, Graduate School of Clinical Dental Science, The Catholic University of Korea, Seoul 06591, Republic of Korea

**Keywords:** cell differentiation, cell survival, gingiva, osteogenesis, stem cells, vitamin E

## Abstract

*Background and Objectives*: Vitamin E is reported to expedite new bone formation in animal models, and this has led to a decrease in the time needed for treatment. In this study, human gingiva-derived stem cell-derived spheroids were examined to determine the effects of vitamin E on cell survival, osteogenic differentiation, and mineralization. *Materials and Methods*: Human gingiva-derived stem cells were used to create spheroids, which were then cultivated with vitamin E at doses of 0, 0.1, 1, 10, and 100 ng/mL. The morphological examination and the qualitative and quantitative vitality of the cells were assessed. Alizarin Red S staining and alkaline phosphatase activity assays were performed on days 7 and 14 to evaluate the osteogenic differentiation. The expression levels of RUNX2 and COL1A1 were assessed using a real-time polymerase chain reaction. *Results*: The addition of vitamin E did not appear to alter the spheroid’s shape at the measured quantities without altering the diameter. During the culture time, the majority of the cells in the spheroids fluoresced green. Regardless of concentration, there were substantial increases in cell viability in the vitamin E-loaded groups on day 7 (*p* < 0.05). On day 14, the Alizarin Red S staining was statistically higher in the 1 ng/mL group compared to the unloaded control (*p* < 0.05). The addition of vitamin E to the culture enhanced the mRNA expression levels of RUNX2, OCN, and COL1A1 based on the real-time polymerase chain reaction data. *Conclusions*: We draw the conclusion that vitamin E may be used to promote the osteogenic differentiation of stem cell spheroids in light of these data.

## 1. Introduction

Vitamin E is a substance frequently found in tocopherols and tocotrienols (α, β, γ, δ) and is well known for its anti-inflammatory, anti-cancer, antioxidant, and anti-bacterial properties [1]. In general, humans cannot produce vitamin E; thus it must be consumed through eating [2]. Nuts and seeds, including sunflower seeds, vegetable oils, safflower oil, spinach, and avocado, are some good sources of vitamin E [3]. Healthy, diabetic, and metabolic syndrome participants all showed improved redox and inflammatory status after taking vitamin E [4]. The number of colonies formed from erythroid colony-forming units grew dramatically as vitamin E levels rose by 75% and 86% compared to the control, respectively, showing that these drugs were roughly comparable in their ability to shield the bone marrow against the toxicity brought on by azidothymidine [5]. Previous reports have shown that a lack of vitamin E reduces bone calcification [6]. Supplementing with palm vitamin E at a greater dose increases the calcium content of the bones [6]. According to animal models, vitamin E is said to increase the growth of new bones, which shortens the length of their healing process [7]. Vitamin E may boost the expression of bone morphogenetic protein-2 when bones are being repaired [8]. Bone quality is enhanced, bone resorption is reduced, and bone production is accelerated by vitamin E [9]. In a previous report, gamma-tocotrienol, the most effective type of vitamin E, increased bone growth in normal rats [10]. Because it controls osteoclast fusion, serum vitamin E was demonstrated to be a factor in determining bone mass [11]. Vitamin E (mixed-tocopherol) supplementation has shown a preventative impact on bone loss in postmenopausal osteopenic women through anti-resorptive activity in a double-blinded, randomized, placebo-controlled trial research [12].

Although there is some evidence to suggest that vitamin E may have some impact on stem cells, there have only been limited studies in this area [13,14,15]. According to one study, mesenchymal stem cells can multiply and differentiate more quickly in vitro when vitamin E is present [13]. Vitamin E and the selenium therapy of mesenchymal stem cells enhanced immunomodulatory effects [14]. Acute kidney injury was treated with vitamin E, and both mesenchymal stem cells from bone marrow and vitamin E had therapeutic benefits, and their combined therapy produced improved results [15]. Pharmacological pretreatment is regarded as a rational approach to harness mesenchymal stem cells that have a higher therapeutic potential, and pretreating mesenchymal stem cells derived from Wharton’s jelly with vitamin E, improves their tolerance to the hostile niche of the fibrotic liver, further increasing their efficacy for hepatic fibrosis [16]. In this way, we hypothesized that vitamin E could be applied to stem cell therapy, including tissue engineering and regenerative medicine. This investigation looked at how vitamin E affected the ability of human mesenchymal stem cell-derived spheroids to differentiate into osteoblasts and mineralize.

## 2. Materials and Methods

### 2.1. Mesenchymal Stem Cells from the Gingiva Used in the Current Study Design and Manufacturing Stem Cell Spheroids

The Institutional Review Board of Seoul St. Mary’s Hospital, College of Medicine, The Catholic University of Korea examined and approved the current study protocol (KC21SASE0225, Approval date: 6 April 2021). Mesenchymal stem cells from gingiva that had multipotent differentiation potential were isolated during periodontal treatment using the previously described techniques [17]. Tissues from the gingiva were de-epithelialized, diced, and enzyme-digested. The stem cells obtained from gingiva were put into a culture dish. The cells were raised at 37 °C in an incubator with 95% air and 5% CO_2,_ and the culture media was changed every one to two days.

Stem cells were seeded at a density of 1 × 10^6^ cells/well in silicon elastomer-based concave microwells with a diameter of 600 µm (StemFIT 3D; MicroFIT, Seongnam-si, Gyeonggi-do, Republic of Korea) and cultured in an osteogenic medium containing an alpha-minimal essential medium (Gibco, Grand Island, NY, USA). The final vitamin E concentrations were 0 ng/mL, 0.1 ng/mL, 1 ng/mL, 10 ng/mL, and 100 ng/mL, respectively. Morphological analysis was performed on days 1, 3, 5, and 7 (CKX41SF, Olympus Corporation, Tokyo, Japan).

### 2.2. Assessing the Quality of Cellular Vitality

Molecular Probes, Eugene, OR, USA, provided a commercially available two-color assay based on plasma membrane integrity and esterase activity on days 1 and 7 [18]. The spheroids were incubated for 60 min at room temperature, and the stem cell spheroids were viewed at ×100 magnification.

### 2.3. Quantitative Analysis of Cellular Vitality

On days 1, 3, 5, and 7, a water-soluble tetrazolium salt-based assay kit (Cell Counting Kit-8, Dojindo, Tokyo, Japan) was used to perform quantitative cellular viability tests [19]. The assay was used to determine which cells were alive. It assessed the ability of mitochondrial dehydrogenases to oxidize water-soluble tetrazolium-8 into a formazan product. With the use of a microplate reader, the spectrophotometric absorbance was measured (BioTek, Winooski, VT, USA). The analysis was conducted using three experimental replicates.

### 2.4. Alkaline Phosphatase Activity and Levels of Calcium Deposition

An anthraquinone dye assay was used to assess osteogenic differentiation on days 7 and 14 in order to gauge the calcium deposits and the level of alkaline phosphatase activity [20]. Cell spheroids grown on culture plates containing osteogenic magnesium were obtained on days 7 and 14. Using a commercial kit, alkaline phosphatase activity was assessed (K412-500, BioVision, Inc., Milpitas, CA, USA). In order to measure the absorbance at 405 nm, cell lysates were added to an assay solution (K412-500; BioVision, Inc.) together with a 5 mM p-nitrophenylphosphate substrate [19]. The combination was then incubated at 40 °C for 30 min. After being cleaned, fixed, and stained for 30 min at room temperature with a 2% Alizarin Red S solution (cat. no. 0223; ScienCell Research Laboratories, Inc.), stem cell spheroids were processed. The bound dyes were then measured for 15 min at 560 nm with 10% cetylpyridinium chloride (cat. no. C0732; Sigma-Aldrich; Merck KGaA, Saint Louis, MO, USA) [21].

### 2.5. Measurement of RUNX2, and COL1A1 mRNA Using Real-Time Quantitative Polymerase Chain Reaction (qPCR) after Total RNA Extraction

The total RNA extraction was carried out using a commercial kit (Thermo Fisher Scientific, Inc., Waltham, MA, USA) in accordance with the manufacturer’s instructions [22]. The RNA quality was evaluated using the RNA 6000 Nano Chip kit from Agilent Technologies, and the RNA amount was evaluated using a spectrophotometer (ND-2000, Thermo Fisher Scientific, Inc.). RNA was used as the reverse transcription template, and a reverse transcriptase was used (SuperScript II; Invitrogen, Carlsbad, CA, USA).

By using qPCR on day 7, the mRNA expression of RUNX2 and COL1A1 was discovered, which are the markers of osteogenic differentiation. Our sense and antisense PCR primer designs were based on GenBank. Listed here are the primer sequences of RUNX2, COL1A1, and β-actin, respectively; the accession numbers were NM 001015051.3, NM_000088.4, and No.: NM 001101, respectively [23,24].

### 2.6. Statistical Analysis

Each value is displayed as the mean minus the standard deviation. Testing for normality and variance equality was conducted. By using a one-way analysis of variance and Tukey’s post hoc test, comparisons between the groups were made. Each analysis included the evaluation of three experimental replicates.

## 3. Results

### 3.1. Mesenchymal Stem Cell-Derived Cell Spheroids from Human Gingiva

**Figure 1:**
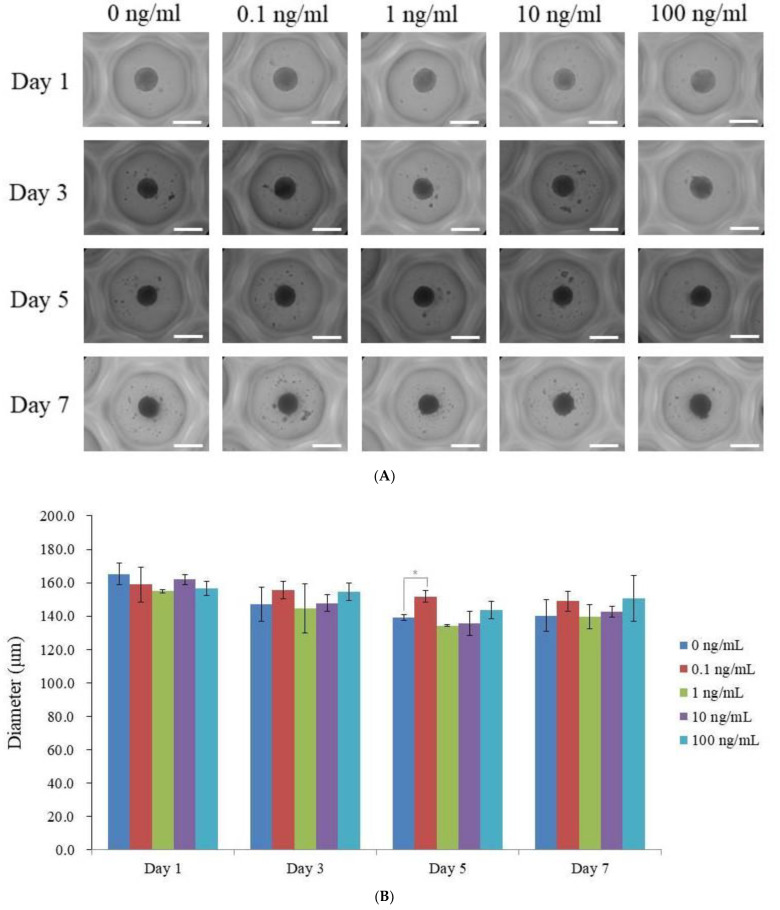
Analyses of morphology. (**A**) Stem cell spheroids’ morphologies on days 1, 3, and 5 and 7 after being exposed to different vitamin E concentrations. A total of 200 μm (with an original magnification of 200) is indicated by the scale bar. (**B**) The stem cell spheroids’ diameters on days 1, 3, 5, and 7. * Day 5 time-matched comparison with the 0 ng/mL group: *p* < 0.05.

Figure 1A depicts the shape of a spheroid treated with vitamin E at final concentrations of 0, 0.1, 1, 10, and 100 ng/mL on days 1, 3, 5, and 7. Regardless of whether vitamin E was applied on day 1, all stem cell spheroids kept their round shape. The morphology of stem cell spheroids did not change throughout the course of seven days. Despite the fact that each stem cell spheroid’s size varied from day 1 to day 7, they all continued to be round (*p* > 0.05). The diameters of vitamin E groups were measured on Days 1, 3, 5, and 7 at various concentrations (0, 0.1, 1, 10, and 100 ng/mL). Figure 1B displays the spheroids’ diameter. In general, the diameters of the vitamin E groups and the control group did not differ significantly from one another (*p* > 0.05). The diameters of the vitamin E groups, however, were noticeably different from the control group on day 5 (*p* < 0.05).

### 3.2. Assessing Cellular Viability Quantitatively and Numerically to Gauge Cellular Vitality

**Figure 2:**
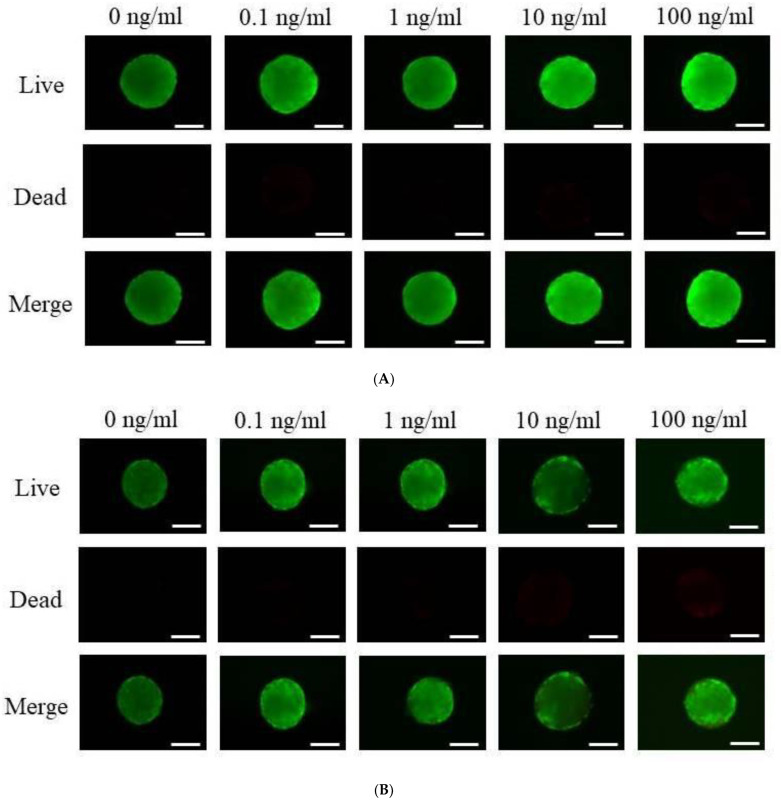

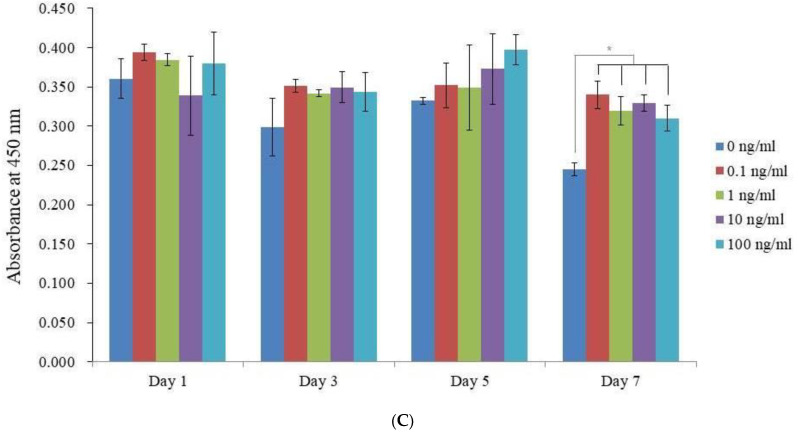
Cellular viability assessment. (**A**) Stem cell spheroids on day 1 with live, dead, and merged cell pictures. (**B**) Stem cell spheroids on day 7 with live, dead, and combined cell pictures. (Original magnification: 100) The scale bar denotes 100 μm. (**C**) Cell viability measured on days 1, 3, 5, and 7 using the Cell Counting Kit-8. * On day 7, *p* < 0.05 compared to the time-matched 0 ng/mL group.

Using a Live/Dead Kit assay, the qualitative viability of stem cells was assessed on days 1 and 7. (Figure 2A,B). The bulk of the stem cells had a spherical shape and intense green fluorescence on day 1, demonstrating their viability (Figure 2A). The cells were incubated for longer on day 7, but there was no discernible decrease in green fluorescence (Figure 2B).

The graph in Figure 2C presents the levels of cellular viability measured on days 1, 3, 5, and 7. The quantitative cellular viability of the vitamin E groups and the control group on days 1, 3, and 5 did not differ significantly from one another (*p* > 0.05). At day 7, there were noticeable changes in the cell viability across the 0.1, 1, 10, and 100 ng/mL groups (*p* < 0.05).

### 3.3. Analyzing the Activity of Alkaline Phosphatase and Alizarin Red S Staining

**Figure 3:**
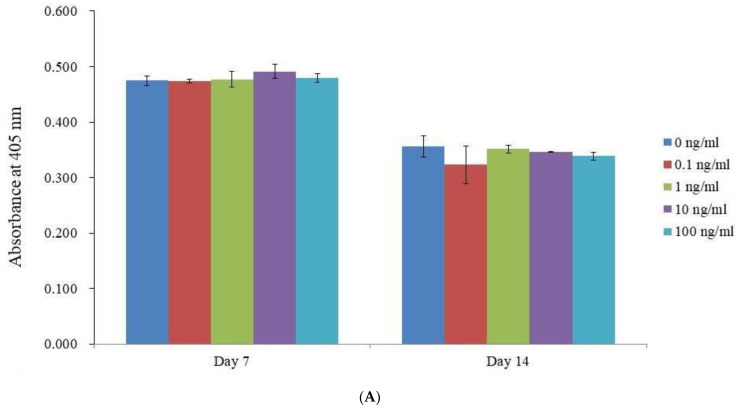

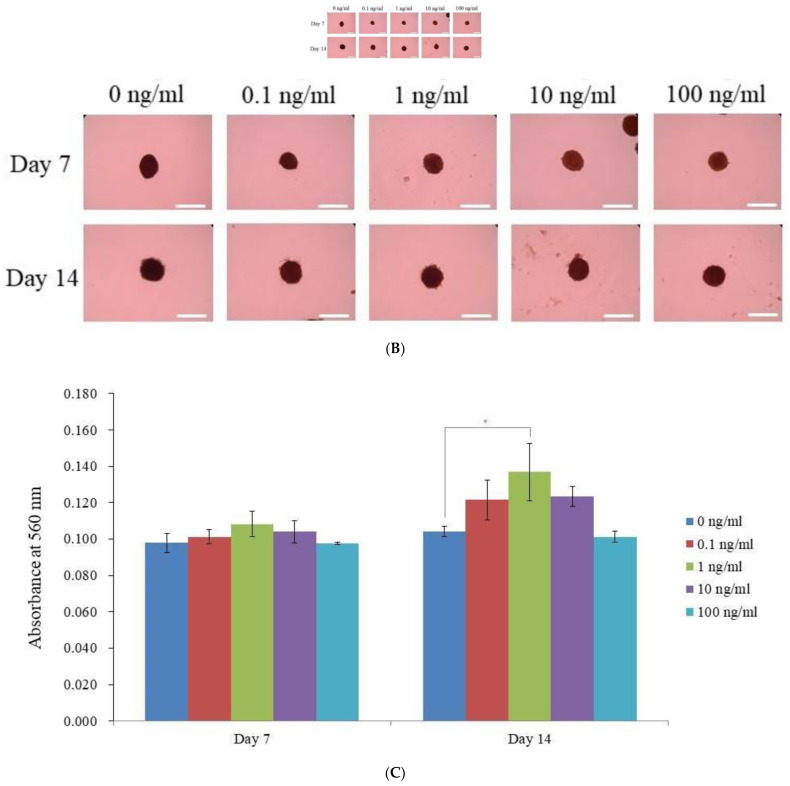
Analyzing the activity of alkaline phosphatase and Alizarin Red S staining. (**A**) Graphical results of alkaline phosphatase activity tests on days 7 and 14. (**B**) Results of Alizarin Red S staining on days 7 and 14. (**C**) Quantitative results and Alizarin Red S staining on days 7 and 14. * *p* < 0.05 vs. time-matched 0 ng/mL group on day 14.

On Days 7 and 14, irrespectively, there was no significant difference in the alkaline phosphatase activity between the vitamin E-loaded groups and the unloaded control (*p* > 0.05) (Figure 3A). Calcium deposits in each group were clearly observed on days 7 and 14. (Figure 3B). When compared to the unloaded control group on Day 14, the Alizarin Red S staining in the 1 ng/mL group was statistically higher (*p* < 0.05) (Figure 3C).

### 3.4. qPCR Analysis of RUNX2 and COL1A1

**Figure 4:**
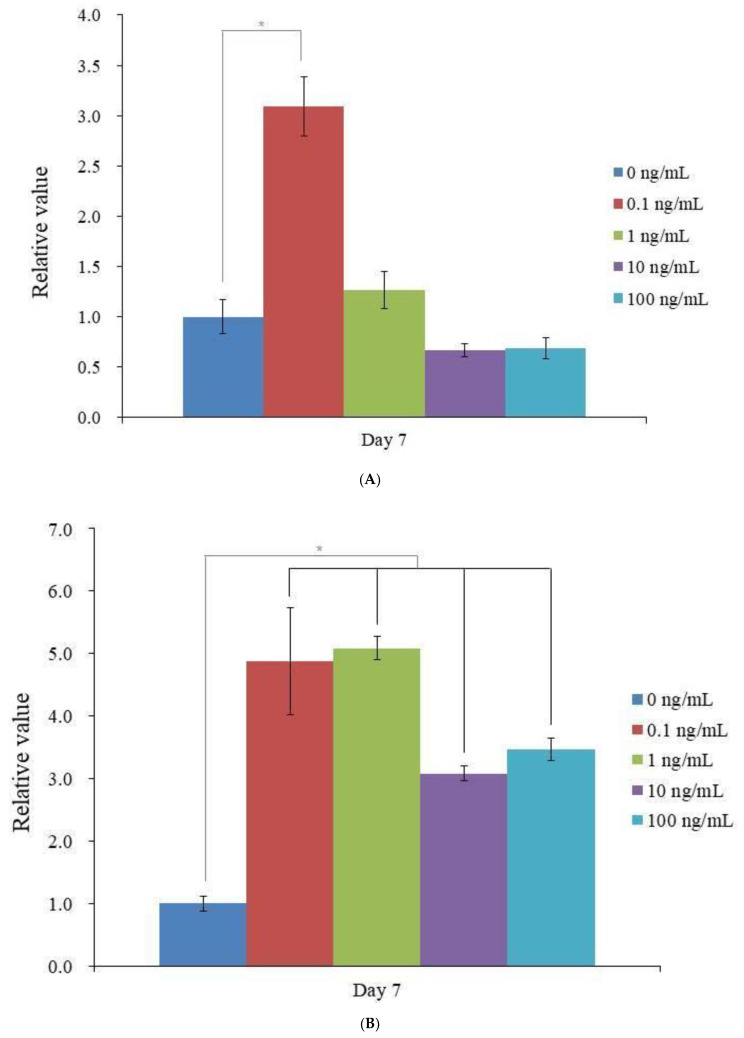
The quantitative expression of mRNA. (**A**). RUNX2 mRNA expression was measured in real time by a polymerase chain reaction on day 7. * *p* < 0.05 in comparison to day 7’s 0 ng/mL. (**B**). Measurement of COL1A1 mRNA expression by real-time polymerase chain reaction on day 7. * *p* < 0.05 in comparison to day 7’s 0 ng/mL.

According to qPCR, RUNX2 mRNA levels showed a significant increase at 0.1 ng/mL of vitamin E supplementation on day 7 (Figure 4A) (*p* < 0.05). The expression of COL1A1 was significantly elevated at 0.1, 1, 10, and 100 ng/mL in the vitamin E groups on day 7 (Figure 4B) (*p* < 0.05).

## 4. Discussion

In this study, the researchers focused on investigating the effects of vitamin E on the differentiation of human mesenchymal stem cells into osteogenic tissue and mineralization. The study utilized various techniques, including alkaline phosphatase activity and real-time quantitative polymerase chain reactions, to identify differentiation into an osteogenic lineage and an increase in mRNA levels of RUNX2 and COL1A1 after administering vitamin E.

It is noteworthy that vitamin E comprises eight structurally different compounds, including alpha, beta, gamma, and delta-tocopherol, as well as alpha, beta, gamma, and delta-tocotrienol [25]. One of the isomers of vitamin E, natural delta-tocotrienol, was found to protect bone marrow and human CD34(+) cells against radiation-induced damage through the extracellular signal-related kinase activation-associated mammalian target of rapamycin survival pathways [26]. Moreover, tocotrienols can help with the symptoms of alpha-tocopherol deficiency [25].

A stem cell spheroid culture was employed in this investigation. Stem cell approaches have attracted a lot of attention, and stem cells have been applied for regenerative medicine [27,28,29,30,31,32,33,34]. By enabling cell–cell and cell–matrix interactions, a spheroid culture technique replicated the physicochemical environment in vivo while overcoming the drawbacks of a conventional monolayer cell culture [35]. Making stem cell spheroids can be conducted using a variety of methods and instruments, such as the hanging drop, bioreactor magnetic levitation, and microwell methods [36]. With the microwell approach, cells are seeded into microwells that are intended to encourage cell aggregation and spheroid development, and this technique enables the creation of spheroids with precise dimensions and forms [37]. Microfabrication methods, such as soft lithography or microcontact printing, can be applied to microwells [38]. Cells in spheroids displayed a robust osteogenic response to the differentiation medium, including the increased mRNA expression of alkaline phosphatase, collagen type I, and osteocalcin compared to those grown in a control medium [39]. The osteogenic potential of periodontal ligament mesenchymal stem cells was said to be enhanced by a spheroid culture, and it has been proposed that spheroid-cultured stem cells may be a novel and practical technique in regenerative medicine [40]. Injectable small-size small adipose-derived stem cell spheroids may be a novel and less invasive therapeutic approach for treating bone abnormalities because they encourage bone regeneration when grown in 3D without a scaffold and under in vitro and in vivo circumstances [41]. In contrast, a different study found that despite a trend toward an improvement in vitro mineralization, constructs made of two- and three-dimensional bone marrow mesenchymal stromal cells were performed similarly in vivo [42]. Bone repair in rat calvarial deformities employed spheroid or dissociated mesenchymal stromal cells in a scaffold-hydrogel structure [42].

This study demonstrated that adding vitamin E to stem cell spheroids improved osteogenic development. According to one study, vitamin E could help bone regeneration in animals with bone abnormalities [43]. The results of the study suggested that vitamin E administration may benefit bone regeneration because it hastened to heal and boost bone production in animals. In an animal model, a different study discovered that vitamin E could enhance the integration of dental implants with bone tissue [44]. The results of this study suggested that the vitamin E coating of dental implants may be useful for implant osseointegration since it enhances the bone–implant interface and increases the amount of bone tissue surrounding the implants. Some research has, however, produced contradictory findings. It was shown that vitamin E reduced bone mass by promoting the fusion of osteoclasts [11]. Previous studies discovered that vitamin E supplementation did not succeed in regaining the fractured bone’s strength with tibial fractures [45]. In a male rat model of osteoporosis, vitamin E supplements did not raise bone marrow density levels [46]. However, when the impact of alpha-tocopherol on cell growth and differentiation was studied, no evidence of enhanced growth or the increased production of extracellular matrix proteins collagen type I, osteonectin, or osteocalcin was found [2]. Pure alpha-tocopherol supplementation did not increase the amount of calcium in the bones [6]. Although there is some evidence that vitamin E may affect bone regeneration, further studies are required to completely comprehend these effects and their potential therapeutic implications.

Interestingly, vitamin E was found to inhibit apoptosis by reducing caspase 3 expression, increasing Bcl 2 expression, and reducing DNA oxidative damage in bone marrow hemopoietic cells at the early stages of steroid-induced femoral head necrosis in rabbit models [47]. This study demonstrated that, at concentrations of 0.1, 1, 10, and 100 ng/mL, vitamin E treatment significantly increased cellular viability when compared to the control. RUNX2 was reported to stimulate proliferation and is necessary for osteoblast progenitor growth [48]. The expression of RUNX2 in the bone and osteogenic front of a suture is essential for cranial suture closure and membranous bone morphogenesis. RUNX2 is a master transcription factor of osteoblast development [49]. After commitment to osteoblasts, RUNX2 is necessary for the expression of the main bone matrix protein genes in an animal model [50]. Preosteoblasts are where the RUNX2 protein was initially discovered, and immature osteoblasts express it at a higher level than mature osteoblasts [51]. One of the osteogenic markers of mesenchymal stem cells is COL1A1 [52]. Osteogenesis imperfecta, characterized by increased bone fragility, may result from COL1A1 mutations [53]. Additionally, the mutated COL1A1 gene in osteogenesis imperfecta was corrected using genome editing [54]. One of the primary components of the organic matrix, COL1A1, can serve as a marker for the deposition of an extracellular matrix [55].

Vitamins may play a dual role in bone health, benefiting bones in the right amounts but potentially harming them in excess doses [56]. Source and dosage significantly affect the effects that are seen, with bioavailability perhaps playing a major role in achieving the desired result [4]. At 1 ng/mL, mineralization had its greatest impact, while at 0.1 ng/mL, genes related to osteogenesis had the greatest expression. Depending on the type of cells, the system model, culture time, and degree of cell differentiation in different doses may be necessary to produce the desired effect [57].

The unique aspect of this work is that it looks at how vitamin E affects cell survival, osteogenic differentiation, and the mineralization of spheroids made from human gingiva stem cells. This study especially investigates the effects of vitamin E on human gingiva-derived stem cells, which are a promising source of stem cells for regenerative medicine. Additionally, the study of cell–cell interactions and the possibility for improved osteogenic differentiation are made possible by the use of stem cell spheroids. Additionally, this study investigated a variety of vitamin E concentrations to identify the ideal dosage and encourage osteogenic differentiation. Overall, this study adds new knowledge to how vitamin E may be used to encourage osteogenic differentiation in stem cell spheroids, which may have implications for upcoming treatments, including regenerative medicine.

While the results of the study are promising, there are some limitations to consider. Human gingiva-derived stem cell–derived spheroids were used in vitro for the investigation. It is vital to keep in mind that the circumstances in vitro may not accurately reflect the complexity of in vivo surroundings [58], even if these findings offer insightful information about the possible impacts of vitamin E on osteogenic differentiation. Further studies using animal models may be needed to validate in vitro studies. Vitamin E’s effects on other osteogenic substances were not compared in this study; this could have revealed important information about the possible benefits of utilizing vitamin E to encourage osteogenic differentiation. This study assessed the impact of vitamin E on osteogenic differentiation on days 7 and 14. Future research should focus on determining the long-term effects of vitamin E on osteogenesis and mineralization.

## 5. Conclusions

According to the results of this study, vitamin E may have the ability to encourage osteogenic differentiation in stem cell spheroids, which has significant implications for tissue engineering and regenerative medicine. The potential of these cell types for tissue engineering and regenerative medicine applications is also highlighted by the utilization of human gingiva-derived stem cells and spheroids in this study. We draw the conclusion that vitamin E may be used to promote the osteogenic differentiation of stem cell spheroids in light of these data.

## Data Availability

All the information generated or analyzed for this study is contained in this publication.
